# Incidence and mortality of incidental prostate cancer: a Swedish register-based study

**DOI:** 10.1038/sj.bjc.6604834

**Published:** 2008-12-16

**Authors:** O Andrèn, H Garmo, L Mucci, S-O Andersson, J-E Johansson, K Fall

**Affiliations:** 1 Department of Urology Örebro University Hospital, Örebro 701 85, Sweden; 2 Regional Oncologic Center, Uppsala University Hospital, Uppsala 75185, Sweden; 3 Channing Laboratory, Harvard Medical School/Brigham and Women's Hospital, Boston, MA 02115, USA; 4 Department of Epidemiology, Harvard School of Public Health, Boston, MA 02115, USA; 5 Department of Medical Epidemiology and Biostatistics, Karolinska Institutet, Stockholm 171 77, Sweden

**Keywords:** incidental prostate cancer, incidence, mortality, transurethral resection of the prostate (TURP)

## Abstract

In a national register-based study of incidence trends and mortality of incidental prostate cancer in Sweden, we found that a significant proportion (26.6%) of affected men diagnosed died of their disease, which challenges earlier descriptions of incidental prostate cancer as a non-lethal disease.

The clinical significance of incidental prostate cancer (PCa) detected by transurethral resection (TURP) or by open adenoma enucleation (OAE) for assumed benign hyperplasia has been a matter of debate (Epstein *et al*, 1986; Epstein, 1992; Bostwick, 1995; Adolfsson, 2008). The main question is whether or not these tumours are biologically different from other prostate cancers diagnosed by needle biopsy. Incidental tumours, i.e. tumours found in the specimens of men undergoing surgery for benign prostatic hyperplasia (BPH), have been regarded as harmless and have sometimes therefore, been left without treatment, especially those of small volume (Cantrell *et al*, 1981). However, recent studies have demonstrated that with increasing tumour volume (>5% of totally resected tissue; T1b tumours), the clinical course of PCa becomes more unfavourable, and is comparable to that of palpable T2 tumours (Andren *et al*, 2006; Cuzick *et al*, 2006; Robinson *et al*, 2007). Understanding the biology of incidental prostate cancer will be increasingly important as results from biomarker studies based on TURP tissue repositories become available.

## Materials and methods

Using nationwide data from the Swedish National Inpatient Register, The Swedish Cancer Register and the Cause of Death Register, linked by national registration number, a unique 10-digit identification number assigned to all Swedish residents, we identified all individuals in the Swedish Inpatient Register who had been discharged after TURP or OAE between 1970 and 2003 (*n*=76 778). There was almost no private institutional care available in Sweden during the study period; essentially all men were referred to and treated at the main hospital in their county of residence. By record linkage to the essentially complete Cancer Registry (Mattsson and Wallgren, 1984), we identified all men who were diagnosed with PCa through TURP or OAE, hereafter referred to as incidental PCa, which required the following criteria to be fulfilled: (1) the date of PCa diagnosis had to follow that of first TURP/OA admission, (2) the date of PCa diagnosis had to be set within 14 days from discharge, (3) the PCa diagnosis had to be histopathologically verified, and (4) the hospital stay had to be less than 60 days. A diagnosis of high-grade intraprostatic neoplasia was not classified as PCa. In total 23 288 men were identified with incidentally diagnosed PCa. For comparison, we also identified all men diagnosed with non-incidental PCa in the Swedish Cancer Register within the same geographical area during the same period. We excluded all autopsy detected PCa. In total, the study population including both incidental and non-incidental PCa comprised 135 492 men.

We calculated the age-standardised incidence rates of PCa using the age distribution of the Swedish population on 1 January 2000, obtained from Statistics Sweden (Statistiska Centralbyrån (Statistics Sweden), 2008). The men were followed prospectively from date of cancer diagnosis to death from cancer, or censored from other causes of death or at end of follow-up (31 December 2003). We obtained information on cause of death through linkage to the Cause of Death Registry, and the international classification of disease (ICD) was used to identify PCa (ICD10=C619) as the main cause of death. We calculated cumulative incidence curves to illustrate the risk of PCa death while accounting for the competing risk of death from other causes (Kalbfleisch and Prentice, 2002). All statistical calculations were performed using the statistical programme package R (Ihaka and Gentleman, 1996).

## Results

**Figure 1:**
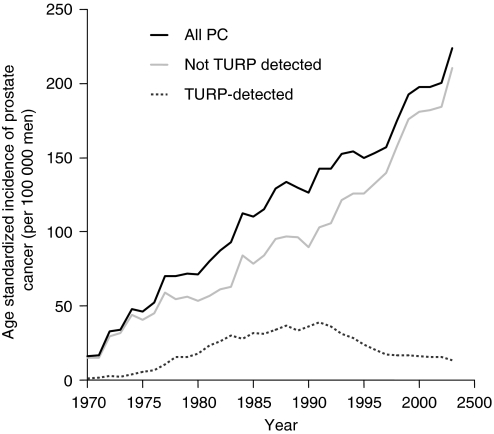
Age-standardised incidence of TURP-detected and total prostate cancer in Sweden between 1970 and 2005.

The characteristics of the study population as well as for all TURP/OAE procedures undertaken in Sweden between 1970 and 2003 are presented in Table 1. The age-standardised incidence of incidental PCa peaked in 1991 at around 37 cases per 100 000 men (Figure 1). Thereafter, it declined successively and reached 12 cases per 100 000 in 2003. During the entire study period, 1970–2003, the rate of total PCa increased continuously, being almost 15 times higher at the end of the study period than at the beginning. The upward trend was initially paralleled by an increased frequency of TURP, but PCa incidence continued to rise after the incidence of incidental PCa had levelled off.

We observed a marked decrease in hospitalisation time over calendar time among men with incidental prostate cancer. In 1970, when the TURP technique was first introduced, the hospitalisation time was comparatively long, about 3 weeks (median 24, interquartile range, 14–33), and after a continuous decrease, it levelled off at 3 days (median 3, interquartile range, 2–5) in the most recent period.

Among men with incidental PCa, 6300 died of PCa, 11 850 died of intercurrent causes, and 5138 were still alive at the end of 2003. The 10-year PCa-specific mortality was 26.6 (95% CI: 26.0–27.2) among men with incidental PCa and 40.7 (95% CI: 40.4–41.0) among men with non-incidental PCa. In the pre-PSA-era (before 1992), the 10-year PCa-specific mortality was 28.2% (95% CI: 27.5–28.9) among men with incidental PCa and 46.3% (95% CI: 45.9–46.8) among men with non-incidental PCa. The corresponding estimates for the PSA-era (1992 and onwards) were 23.2% (95% CI: 22.2–24.3) among men with incidental PCa and 35.1% (95% CI: 34.6–35.7) among men with non-incidental PCa.

**Figure 2:**
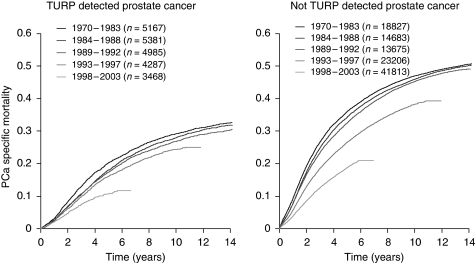
Prostate cancer-specific mortality in Sweden between 1970 and 2003 among men with incidental (left panel) and non-incidental prostate cancer (right panel), by calendar time.

The disease-specific mortality was lower among men with incidentally detected tumours than among men with non-incidental PCa, but a considerable number of PCa-specific deaths occurred in both groups (Figure 2). There was a clear pattern of decreasing mortality in more recent calendar periods in both groups, but even in the most recent time period more than 3400 men with incidental PCa died of their disease.

## Discussion

In this nationwide, population-based study, we observed dramatic changes in the incidence of incidental PCa in Sweden over the last three decades. We found that a significant number of men with incidental PCa die from their disease. These data do not fit earlier descriptions of incidental PCa as a non-lethal disease (Cantrell *et al*, 1981; Epstein *et al*, 1986), but lend support to the hypothesis that incidental PCa have a similar biology to that of non-incidental PCa.

The introduction of TURP in the late 1970s was accompanied by an increase in the incidence of prostate cancer (Haapiainen, 1986). TURP specimens typically represent the transition zone of the prostate (McNeal *et al*, 1988a, 1988b), but tumours detected through TURP may also capture peripheral zone tumours that have grown into the transition zone (Bostwick, 1995). Tumours originating in the transition zone have been viewed as low grade tumours with low potential of malignancy, and men with this type of tumour have often been treated conservatively (Cantrell *et al*, 1981; Epstein *et al*, 1994; Albertsen *et al*, 2005). Nevertheless, 8–37% of all incidental tumours have been shown to ultimately progress (Bostwick, 1995). Evidence from a study on radical prostatectomy patients also indicates that time to biochemical failure is independent of zonal origin of the tumour (Chun *et al*, 2007). These data, as well as our finding of a high disease-specific mortality among men with incidental PCa, support the notion that incidental and non-incidental PCa may share tumour biology and potential for progression.

Our data confirm earlier observations of a declining incidence of incidental tumours after the introduction of PSA screening (Mai *et al*, 2000); incidental PCa currently constitutes around 10% of all diagnosed PCa in Sweden (Regionalt Onkologiskt Centrum ROC, 2003). Our data also demonstrated a decrease in Pca-specific mortality over time. An increased lead time between diagnosis and death after the introduction of the PSA test as well as potentially improved treatments seem more likely to explain this observation than a gradual shift in tumour biology/aggressiveness.

This large, register-based study with an essentially complete follow-up allowed a comprehensive examination of incidence and mortality trends in Sweden over the last three decades. As health care is free to all Swedish residents, selection forces associated with the TURP procedure should be minimal. A limitation, shared by all register-based studies, though, is the quality of available data. The accuracy of the official causes of death data, with regard to prostate cancer, should however be adequate (Fall *et al*, 2008).

These data demonstrated that a significant proportion of men with incidental prostate cancer during the last three decades died from their disease. Findings from biomarker studies that utilise archival tissue specimens from such incidental cases (for prediction of PCa outcomes) may thus contribute to the understanding of the natural course of prostate cancers detected today.

## Figures and Tables

**Table 1:** Incidental prostate cancer, non-incidental prostate cancer and total number of transurethral resections/open adenoma enucleations^a^ among men in Sweden, 1970 and 2003

	**Incidental prostate cancer**		**Non-incidental prostate cancer**		**TURP or OAE**	
	***N***	**%**	***N***	**%**	***N***	**%**
*Age*						
<55	123	(0.5)	2462	(2.2)	708	(0.9)
55–64	2157	(9.3)	17740	(15.8)	8931	(11.6)
65–74	9085	(39.0)	43060	(38.4)	30854	(40.2)
75–84	10114	(43.4)	40769	(36.3)	31163	(40.6)
85+	1809	(7.8)	8173	(7.3)	5122	(6.7)
						
*Years*						
1970–79	2627	(11.3)	13045	(11.6)	12324	(16.1)
1980–86	6693	(28.7)	17116	(15.3)	20793	(27.1)
1987–90	4961	(21.3)	13311	(11.9)	14409	(18.8)
1991–93	3349	(14.4)	12480	(11.1)	10106	(13.2)
1994–98	2795	(12.0)	20270	(18.1)	9790	(12.8)
1999–2003	2863	(12.3)	35982	(32.1)	9356	(12.2)
						
*Time in hospital*						
1–3 days	5675	(24.4)	NA	NA	17929	(23.4)
4–7 days	10997	(47.2)	NA	NA	34758	(45.3)
8–14 days	4175	(17.9)	NA	NA	14964	(19.5)
2–4 weeks	1844	(7.9)	NA	NA	6655	(8.7)
4+ weeks	592	(2.5)	NA	NA	2423	(3.2)
						
*Procedures (N*=)^a^						
0	0	(0.0)	82389	(73.4)	NA	NA
1	18007	(77.3)	23689	(21.1)	NA	NA
2	4118	(17.7)	4719	(4.2)	NA	NA
3	864	(3.7)	1062	(0.9)	NA	NA
4+	299	(1.3)	345	(0.3)	NA	NA
						
*Status at end of study*						
Alive	5138	(22.1)	39527	(35.2)	NA	NA
Death from other causes	11850	(50.9)	35469	(31.6)	NA	NA
Death from CaP	6300	(27.1)	37208	(33.2)	NA	NA
						
*Follow up*						
Time in years, mean (s.d.)	6.1	(4.7)	4.3	(4.0)	NA	NA

a Out of 76 778 procedures, 4456 (5.8%) were open adenoma enucleations and 72 322 transurethral resections of the prostate.
